# Transcriptomics and Proteomics Reveal the Cellulose and Pectin Metabolic Processes in the Tension Wood (Non-G-Layer) of *Catalpa bungei*

**DOI:** 10.3390/ijms21051686

**Published:** 2020-03-01

**Authors:** Yao Xiao, Fei Yi, Juanjuan Ling, Zhi Wang, Kun Zhao, Nan Lu, Guanzheng Qu, Lisheng Kong, Wenjun Ma, Junhui Wang

**Affiliations:** 1 State Key Laboratory of Tree Genetics and Breeding, Key Laboratory of Tree Breeding and Cultivation of State Forestry Administration, Research Institute of Forestry, Chinese Academy of Forestry, Beijing 100091, China; xiaoyao6703@163.com (Y.X.); yifei_yc@163.com (F.Y.); 18829351306@163.com (J.L.); wangzhi6666@126.com (Z.W.); ln_890110@163.com (N.L.); mwjlx.163@163.com (W.M.); 2 Luoyang Academy of Agriculture and Forestry Science, Luoyang 471002, China; 13598181517@163.com; 3 State Key Laboratory of Tree Genetics and Breeding, Northeast Forestry University, Harbin 150040, China; quguanzheng@hotmail.com; 4 Department of Biology, Centre for Forest Biology, University of Victoria, 3800 Finnerty Road, Victoria, BC V8P5C2, Canada; lkong@uvic.ca

**Keywords:** tension wood, transcriptome, proteomics, Raman spectroscopy, cellulose, pectin, *Catalpa bungei*

## Abstract

*Catalpa bungei* is an economically important tree with high-quality wood and highly valuable to the study of wood formation. In this work, the xylem microstructure of *C. bungei* tension wood (TW) was observed, and we performed transcriptomics, proteomics and Raman spectroscopy of TW, opposite wood (OW) and normal wood (NW). The results showed that there was no obvious gelatinous layer (G-layer) in the TW of *C. bungei* and that the secondary wall deposition in the TW was reduced compared with that in the OW and NW. We found that most of the differentially expressed mRNAs and proteins were involved in carbohydrate polysaccharide synthesis. Raman spectroscopy results indicated that the cellulose and pectin content and pectin methylation in the TW were lower than those in the OW and NW, and many genes and proteins involved in the metabolic pathways of cellulose and pectin, such as galacturonosyltransferase (*GAUT*), polygalacturonase (*PG*), endoglucanase (*CLE*) and β-glucosidase (*BGLU*) genes, were significantly upregulated in TW. In addition, we found that the MYB2 transcription factor may regulate the pectin degradation genes *PG1* and *PG3*, and ARF, ERF, SBP and MYB1 may be the key transcription factors regulating the synthesis and decomposition of cellulose. In contrast to previous studies on TW with a G-layer, our results revealed a change in metabolism in TW without a G-layer, and we inferred that the change in the pectin type, esterification and cellulose characteristics in the TW of *C. bungei* may contribute to high tensile stress. These results will enrich the understanding of the mechanism of TW formation.

## 1. Introduction

Wood is a natural and renewable material that is formed by the secondary growth of trees. It is irreplaceable in the fields of construction, paper making, furniture manufacturing and new energy. Wood formation, namely, the process of the differentiation of secondary xylem, is a complex biological process that can be briefly summarized as cambium division, cell enlargement, secondary wall deposition, and programmed cell death to form mature secondary xylem [1]. In particular, the structure and internal metabolism of xylem undergoes substantial changes under long-term pressure from the external environment. This particular xylem, which maintains the balance of growth in trees, is usually called reaction wood [2]. Conifers and broadleaved trees harbor unique types of reaction wood. For conifers, the reaction wood generally exists at the bottom of the bent trunk and is called compression wood [3], while for broadleaved trees, it is located at the top of the curved trunk and is called tension wood (TW) [4]. Early studies have shown that the secondary walls of some trees include a gelatinous layer (G-layer) with a large number of fibers [5,6,7], which was considered the main anatomical feature of TW. Later studies rejected this view because most tropical trees and Magnoliaceae trees do not form a distinct G-layer [8,9].

Plant cell walls are mainly composed of cellulose, hemicellulose, pectin polysaccharide, lignin and a small amount of cell wall proteins [10,11]. Because the structural characteristics and metabolic chemical composition of TW are significantly different from those of normal wood (NW), TW plays an important role in revealing the relationship between cell wall chemical composition and wood properties. The cellulose content in the cell walls of TW was higher than that in the cell walls of NW, especially the G-layer, which contains a large amount of highly crystalline cellulose [12]. Foston, et al. [13] also found that the cellulose crystallinity of TW in poplar (*Populus tremula* × *P. alba*) was higher than that of NW and opposite wood (OW) via Raman scattering spectroscopy. Gierlinger and Schwanninger [14] compared the chemical imaging results for the G- and S-layers of TW in poplar (*P. nigra* × *P. deltoids*) by confocal Raman spectroscopy and found that there was no lignin or other aromatic compounds in the G-layer. In addition to cellulose and lignin, the cell wall also contains a large number of non-cellulosic polysaccharides (hemicellulose and pectin), which play an important role in the formation and development of the cell wall. Pectin has a variety of roles in plant development, such as participating in cell wall formation and support in plants and affecting cell wall surface electrification, porosity and pH [15]. Furthermore, pectin oligosaccharides could reduce cell wall lignification [16].

There are many types of pectin, depending on the side chain. Changes in the content and type of pectin can directly or indirectly influence the cell wall properties. A large amount of RG-I pectin has been found in compression wood and TW [17,18]. In the early stage, the side chain of RG-I pectin, (1-4)-galactan, was found to exist in only the junction of the TW secondary wall and G-layer in poplar [19]. Recent studies have shown that this pectin is also highly enriched in the G-layer of TW [18]. However, homogalacturonan (HG) is mostly present in the middle cellular lamella [20,21] and regulates cell adhesion. Gritsch, et al. [6] found that de-esterified HG was closely associated with TW, often with the G-layer itself. It has also been detected in G-fibers in TW of *Liquidambar styraciflua* [22]. Some reports have also indicated the genes related to pectin metabolism are differentially expressed during TW formation. Andersson-Gunnerås, et al. [12] compared the transcriptomic expression profiles of TW and NW in hybrid poplar and found that several pectin lyase genes (*PL*s) were highly expressed in TW. In addition, one pectin acetylesterase gene (*PAE*) and three *PLs* also exhibited high and significantly low expression, respectively, in *Eucalyptus* TW [23]. All the above studies suggest that pectin contributes greatly to the TW formation and is the one of the main polysaccharides responsible for the properties of TW. A clear understanding of the changes in pectin metabolism that occur during TW formation will provide new insight into the relationship between the chemical and physical properties of wood.

The effects of other macromolecular polysaccharides for cell wall properties was often neglected in the studies of wood formation, because the pulpwood focus cellulose and lignin improvement. What is more, most studies on TW have focused on G-layer species because poplar is a model tree and maintains a G-layer, few studies have been conducted on TW without a G-layer. *Catalpa bungei* is native to China and has high-quality timber [24]; thus, it is an important timber tree and a good material for genetic improvement of wood properties. Based on our previous study, there is no obvious G-layer in TW of *C. bungei*. In this work, we revealed the mechanisms of cellulose and pectin polysaccharide metabolism in fiber cell walls during TW formation in *C. bungei* by combining RNA-seq, proteomics and Raman spectroscopy. This approach enhanced our understanding of the mechanism of TW formation, especially for non-G-layer TW.

## 2. Results

### 2.1. Anatomical Morphology of Different Types of C. bungei Wood

**Figure 1:**
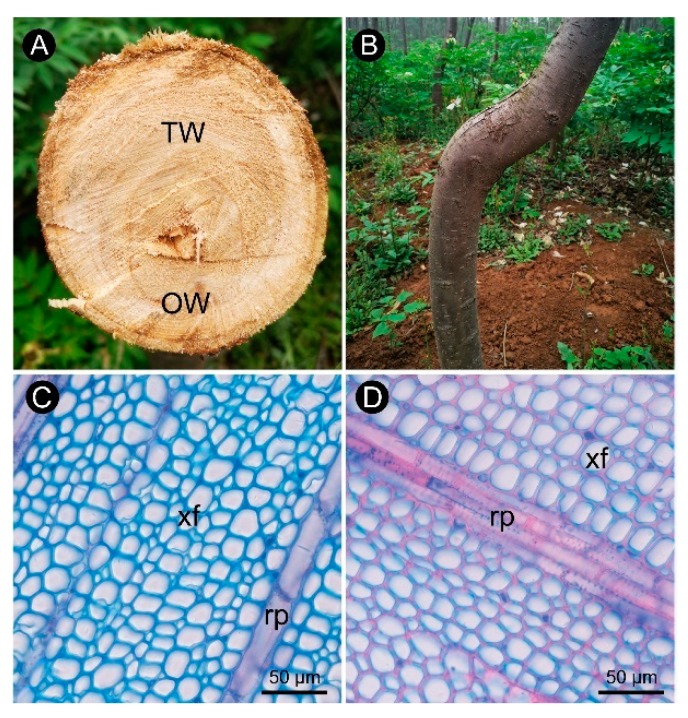
Anatomical morphology of *C. bungei* tension wood naturally bent for 7 years. (**A**) Wood cross-section of bent *C. bungei*. (**B**) Bent *C. bungei*. (**C**) Anatomical morphology of tension wood. (**D**) Anatomical morphology of opposite wood. (xf) Xylem fiber. (rp) Ray parenchyma.

**Figure 2:**
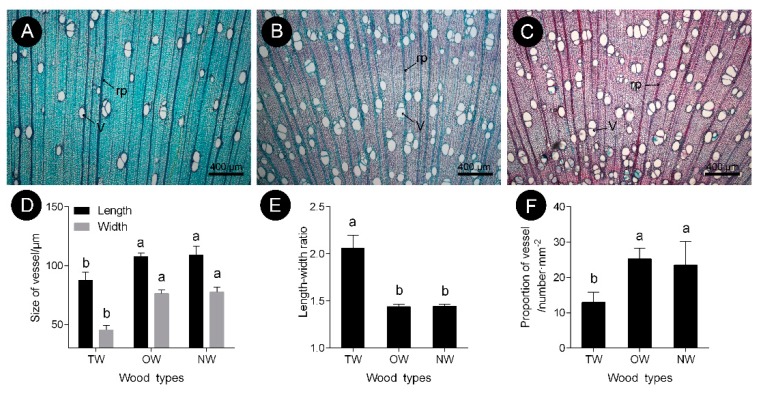
Vessel characteristics of *C. bungei* tension wood that was artificially bent for 3 months. (**A**) Tension wood. (**B**) Opposite wood. (**C**) Normal wood. (**D**) Size of vessel of different wood types. (**E**) Length-width ratio of different wood types. (**F**) Propotion of vessel of different wood types. (V) Vessel. (rp) Ray parenchyma. Different letters indicate significant differences.

The observations of xylem slices of *C. bungei* showed that no G-layer was present in the TW induced by either natural bending or artificial bending (Figure 1 and Figure 2). There were significant differences in the anatomical characteristics between TW and OW and between TW and NW (Figure 2A–C). After dyeing, the TW became dark green, and the OW and NW appeared purplish red. The vessel length and width of the TW were significantly smaller than those of the OW and NW in the early stage, but the length-to-width ratio of the vessels in the TW was significantly larger (by approximately 43%) than that of the vessels in the OW and NW (Figure 2E). In addition, the number of vessels in the TW was significantly reduced (Figure 2F).

**Figure 3:**
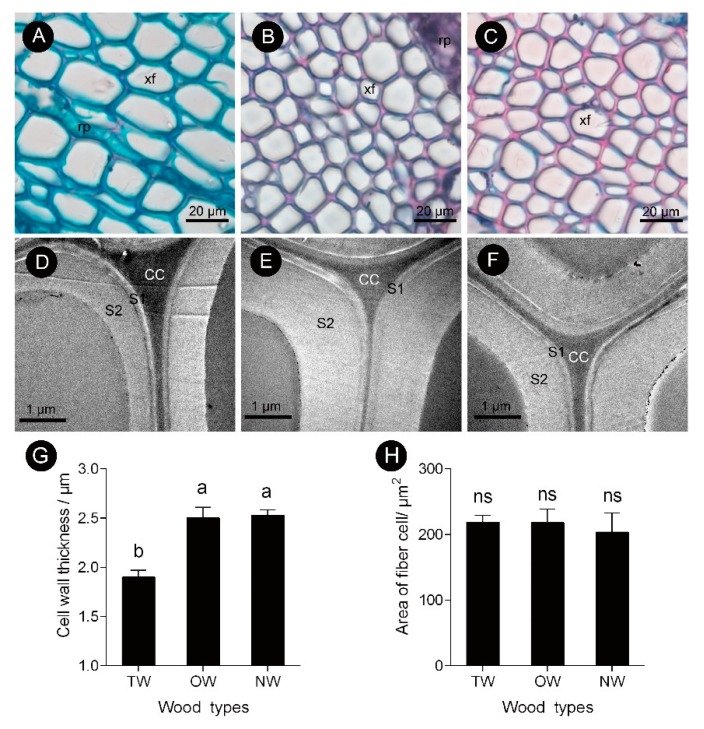
Fiber cell characteristics of *C. bungei* tension wood that was artificially bent for 3 months. (**A** and **D**) Tension wood. (**B** and **E**) Opposite wood. (**C** and **F**) Normal wood. (xf) Xylem fiber. (**G**) Cell wall thickness of different wood types. (**H**) Area of fiber cell of different wood types. (rp) Ray parenchyma. (CC) Cell corner. (S1) First secondary cell wall. (S2) Second secondary cell wall. Different letters indicate significant differences; ns: no significant differences.

This finding showed that the pattern of tracheary element differentiation changed during the formation of TW. There was no significant difference in the size of fiber cells between the TW and OW or TW and NW. However, the secondary wall thickness of TW was significantly reduced by approximately 33% (Figure 3D,G). To further analyze the key factors that contribute to the formation of TW, transcriptome and metabolite analyses were performed.

### 2.2. Identification and Characterization of mRNA and Protein In Different Types of Wood in C. bungei

The 150-bp paired-end sequencing of raw reads was performed by using the Illumina HiSeq 4000 platform. High-quality clean reads were prepared for transcription statistics after filtering out reads that contained adapters, reads with N > 10% and all A bases and low-quality reads (number of bases Q ≤ 20 accounted for more than 50% of the whole reads). The results indicated that a total of 20,489 known transcripts and 17086 new transcripts were found in the samples. More than 71% of the transcripts of each sample mapped to the genome (Table S1).

The labeled peptides were mixed in equal quantities and reversely pre-separated at high pH. The pre-separated components were analyzed by liquid-mass spectrometry (nano-HPLC-MS/MS) at low pH. The data obtained by mass spectrometry were used for protein identification. A total of 31,932 Unique spectra, 17,548 Unique peptides and 5366 proteins were identified (Table S2). The molecular weight of the protein was mainly concentrated in 30–50 kDa (Figure S1), and most of the identified peptides contained 8-11 amino acids (Figure S2). The coverage of peptides in proteins is mainly 5–15% (Figure S3).

### 2.3. DEGs and DEPs Between Different Wood Types

**Figure 4:**
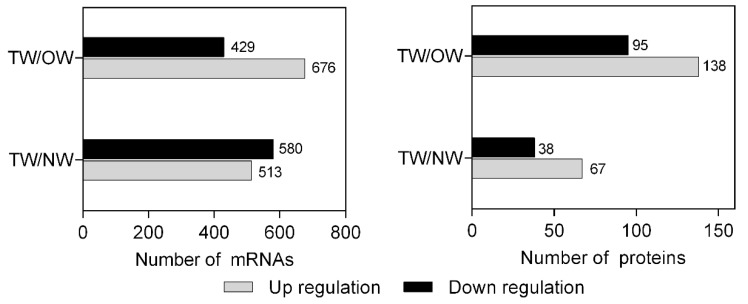
The number of DEGs and DEPs in different wood types.

A total of 513 significantly upregulated mRNAs and 580 significantly downregulated mRNAs were identified in TW compared with NW. A total of 676 mRNAs were significantly upregulated and 429 were significantly downregulated in TW compared with OW (Figure 4). There were 105 DEPs in TW/NW, of which 67 were significantly upregulated and 38 were significantly downregulated. A total of 138 upregulated and 95 downregulated proteins were detected with significantly different expression levels in TW/OW (Figure 4).

**Figure 5:**
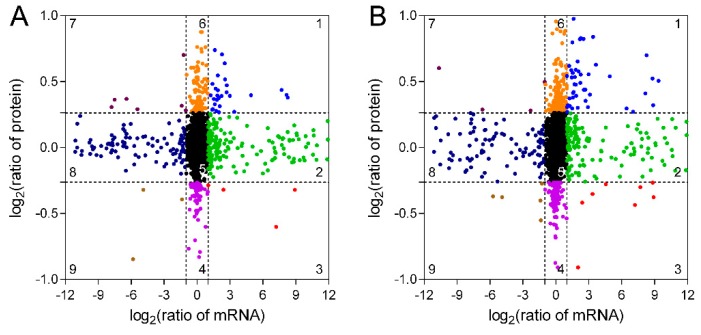
Relationship between mRNA and protein expression in TW/NW (**A**) and TW/OW (**B**).

mRNA and protein expression pattern analysis revealed that 29 and 47 genes in TW/NW and TW/OW, respectively, were upregulated at both in transcription and protein levels (Figure 5, Tables S3 and S4), and most of these genes were attributed to primary and secondary metabolite synthesis pathways. The genes in quadrant 2 and 3 have high transcription levels in TW, among which the protein level in quadrant 2 shows no significant difference between different wood types, and the protein expression level in quadrant 3 is low in TW, and both genes in quadrant 2 and 3 may be subject to post-transcriptional regulation. Genes and proteins in quadrant 9 are both downregulated in TW, and most of these genes are involved in metabolic processes (Tables S3 and S4). Genes in quadrant 1 and 9 may be the key genes associated with the change in chemical composition of TW.

### 2.4. GO Analyses of DEGs and DEPs in Different Wood Types

The GO database was used to resolve the functions of DEGs and DEPs. The results showed that most of the differentially expressed mRNAs in the TW/NW comparison were enriched in the carbohydrate metabolic process. Within the molecular function and cellular component categories, and the functions of differentially expressed mRNAs were mainly related to transporter activities and membranes. This suggests that carbohydrate synthesis and transmembrane transport of metabolites may be among the key steps for the formation of TW. In the TW/OW comparison, most of the GO functional annotations of the differentially expressed mRNAs were enriched in terms in the molecular function category involved in catalytic, hydrolase and transporter activities. Additionally, some genes were also significantly enriched in the carbohydrate metabolic process. This suggests that polysaccharide metabolism is an important process during the early formation of TW.

The GO enrichment results showed that some of the DEGs in TW/OW were involved in the synthesis of cellulose and the construction of the cytoskeleton (Table S5). During the TW formation in *C. bungei*, the DEPs are also involved in carbohydrate metabolism-related functions, such as poly-galacturonase activity, β-fructofuranosidase activity, α-glucosidase activity, carbohydrate derivative transporter activity and extracellular polysaccharide biosynthetic process. In addition, differential protein functions were observed for protein complexes and proteins binding to DNA, most of which were annotated as protein complex assembly, protein-DNA complex, and protein complex subunit organization. This suggests that protein complexes are the forms that are functional during TW formation.

### 2.5. KEGG Pathway Analyses of DEGs and DEPs in Different Wood Types

**Figure 6:**
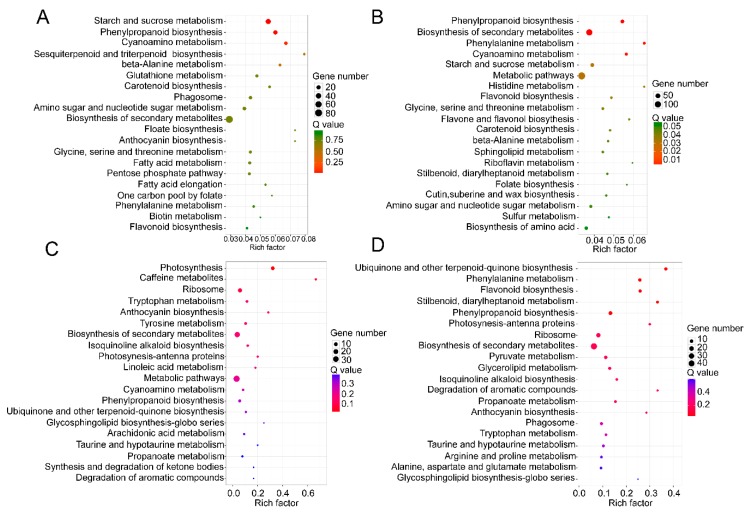
Top 20 of KEGG pathway enrichment of DEGs in the transcriptome (**A** and **B**) and DEPs in the proteome (**C** and **D**). (**A** and **C**) TW/NW, (B and D) TW/OW. The *q* value is the *p* value corrected by false discovery rate method.

The KEGG pathway database was utilized to facilitate the interpretation of the metabolic processes that differed between TW and NW and between TW and OW. Most differentially expressed mRNAs participate in the carbohydrate metabolism, synthesis of secondary metabolites, and phenylpropanoid, sesquiterpenoid, triterpenoid, anthocyanin and flavonoid biosynthesis. Similar to the GO enrichment, most of the differentially expressed mRNAs act in the carbohydrate metabolism pathway, for example “starch and sucrose metabolism” and “amino sugar and nucleotide sugar metabolism”, in both the TW/NW and TW/OW comparisons. (Figure 6).

### 2.6. Changes in Gene and Protein Expression in Cellulose, Hemicellulose and Pectin Biosynthesis during TW Formation

**Figure 7:**
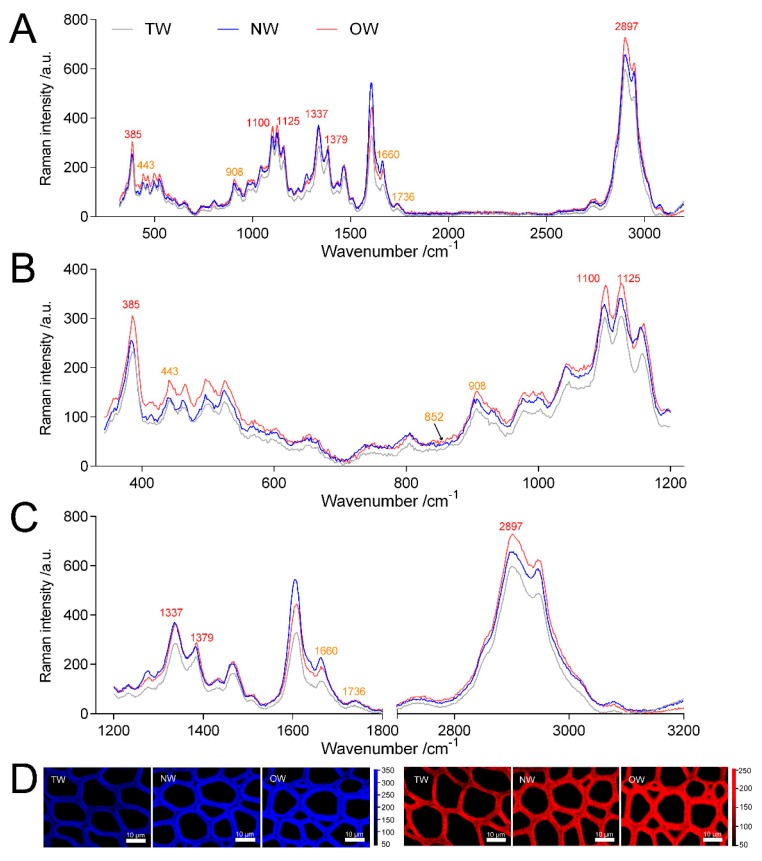
Analysis of Raman spectra of the secondary walls of different wood types. (**A**) Averaged Raman spectra in the range of 350~3200 cm^−1^ of the secondary walls of different wood types. (**B**) Averaged Raman spectra in the range of 350~1200 cm^−1^ of the secondary walls of different wood types. (**C**) Averaged Raman spectra in the range of 1200~3200 cm^−1^ of the secondary walls of different wood types. (**D**) confocal Raman spectral imaging; blue indicates images of xylem calculated by integrating from 2804~2930 cm^−1^, and red indicates images of xylem calculated by integrating from 326~405 cm^−1^.

Figure 7 shows that the Raman intensity of TW and NW were similar and significantly lower than OW in the wavenumbers 320~650 cm^−1^. And the peak value of TW was significantly lower than that of OW and NW in the wavenumbers 900~1800 cm^−1^ and 2000~3200 cm^−1^. In the Raman spectra, the bands observed in the 2800~3000 cm^−1^ range could be assigned to the C-H stretching vibrations of –CH, -CH2, -CH3 groups [25,26,27,28,29], in this region all cell walls compounds contributed to the Raman signal. And the peak of TW was lower than that of OW and NW in the range of this wavelength. Confocal microscopy imaging results also showed that the TW Raman signal was weakest and that the precipitation of cell wall polymers from TW was greatly reduced (Figure 7A).

The wavenumbers 326~405 cm^−1^, 1092~1122 cm^−1^ and 2800~3000 cm^−1^ in the Raman spectra reflect the symmetric bending vibration of CCC in the pyranoid ring, the asymmetric and symmetric stretching vibration of COC in the glucosidic bond and the stretching vibration of CH and CH2 in cellulose, respectively [30,31]. Thus, these characteristic peaks represent the relative cellulose content. The spectral results showed that the TW had the lowest absorption peak at 385, 1100, 1125 and 2897 cm^−1^ (Figure 7).

**Figure 8:**
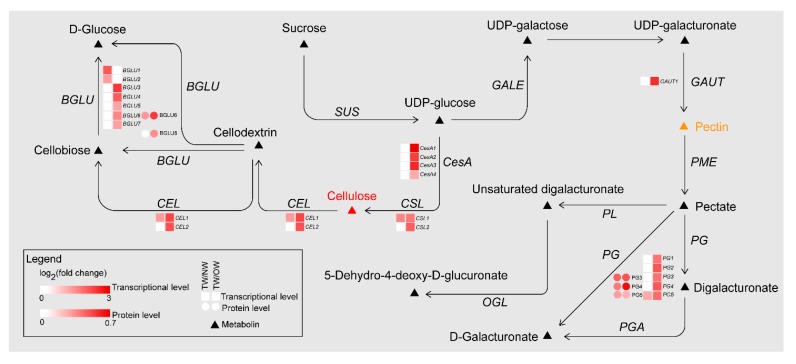
DEGs and DEPs involved in cellulose and pectin biosynthesis pathways of TW formation.

Meanwhile, during the TW formation process, two cellulose catabolism genes (*CELs*) were significantly upregulated in TW/NW and TW/OW, respectively. Two β-glucosidase (*BGLU*) genes were significantly upregulated in TW/NW, and five *BGLU* genes were significantly upregulated. In addition, two BGLU proteins were also found to be significantly upregulated in the TW formation process according to proteomics. Interestingly, not only *BGLU6* transcription but also BGLU6 protein expression in TW was significantly higher than that in OW (Figure 8, Tables S6 and S7). BGLU is one of the members of the cellulase family, hydrolyzing cellobiose to two molecules of glucose. This may contribute to decreased deposition of TW fiber cell wall. We also found that mRNAs related to hemicellulose biosynthesis were differential expression between TW and OW, among which 5 xyloglucan glycosyltransferase (*XTH*) genes and 1 glucomannan 4-β-mannosyltransferase (*CLSA9*) gene were significantly up-regulated in TW (Table S6). This suggested that there may be higher content of xyloglucan and glucomannan in TW.

The polygalacturonase (*PG*) enzyme-coding gene, which is involved in catalyzing pectin degradation in TW, was significantly upregulated in TW compared with NW and OW. There are 5 *PGs* that are upregulated during TW formation, and the protein products of three of these genes (evm.model.group11.687, evm.model.group7.2243 and evm.model.group7.2616) are also present at high levels in TW (Figure 8, Tables S6 and S7). According to the literature [25,26,27,28]. The analysis of the spectra of plant samples in the 1600~1800 cm^−1^ range is used to characterize pectin in plant material. In this study, the two peaks (1660 and 1736 cm^−1^) of TW were lower than that of OW and NW (Figure 7C). This confirmed that pectin in TW was less and may be rapidly degraded. On the other hand, in the cell wall pectin with a different degree of methylesterification can be found. The most prominent Raman marker band for the identification of pectin polysaccharides is centered at 852 cm^−1^ which is due to the vibrations of α-glycosidic bonds in pectin. The wavenumber position of this band is shifted from 858 cm^−1^ for a low methylesterification degree to around 842 cm^−1^ for a high methylation degree [28]. But in our study, there were no obvious absorption peak at 852 cm^−1^, and the signal intensity of TW was lowest at this wavenumber. It implied the lowest methylation degree of TW. In addition, the significant upregulation of the galacturonic transferase gene (*GAUT*), glucuronoxylan glucuronosyltransferase gene (*IRX7*) and glucuronoxylan 4-*O*-methyltransferase gene and protein in the TW/OW or TW/NW comparisons also implied that pectin metabolism was very important for TW formation (Tables S6 and S7). Due to these enzymes can catalyze the biosynthesis of different types of pectin.

**Figure 9:**
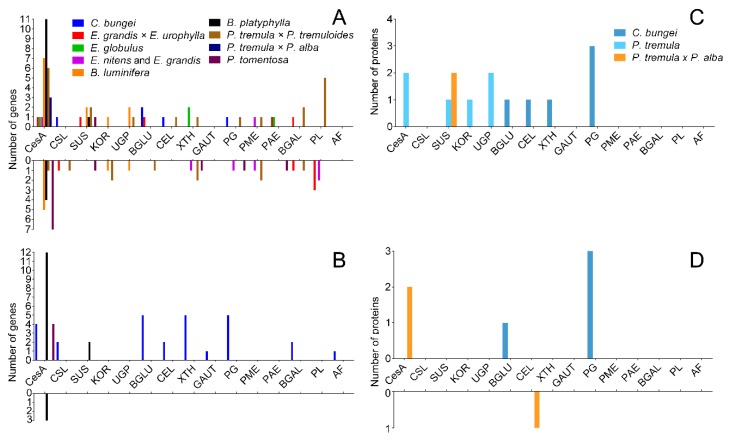
The number of DEGs and DEPs involved in cellulose and pectin biosynthesis during TW formation in different tree species. (**A** and **B**) DEGs in the transcriptome, (**C** and **D**) DEPs in the proteome.

TW has a variety of morphological characteristics, and the most obvious difference is the presence of the G-layer. We reviewed several papers [12,23,32,33,34,35,36,37,38,39] to explore the causes of the non-G-layer TW formation. The expression of cellulose and pectin metabolic genes in *C. bungei* and other trees was compared (Figure 9). The results are shown in Figure 9A,B. A large number of genes related to cellulose synthesis, for instance *CesA* and *SUS*, were upregulated in TW with a G-layer. However, the many cellulase genes (*BGLUs* and *CELs*) were significantly upregulated in *C. bungei* T. For pectin metabolism, some *GAUT*, *PG*, *BGAL* and *AF* genes were highly expressed in TW/OW of *C. bungei*. And *PME*, *PAE* and *PL* were differently expressed in TW of other tree species.

Comparison of the proteome between poplar and *C. bungei* TW formation showed (Figure 9C,D) that several CesA and SUS proteins, which participate in cellulose biosynthesis, had high levels in poplar TW, but no such result was observed in the *C. bungei* TW proteome. Cellulose hydrolysis-related proteins (BGLU and CEL) and pectin degradation-related proteins (PG) were highly expressed in *C. bungei* TW. Differences in cellulose and pectin metabolism could be an important reason for the difference in TW morphology between *C. bungei* and other tree species.

### 2.7. Transcript Levels and qRT-PCR Validation of mRNA in Different Wood Types

**Figure 10:**
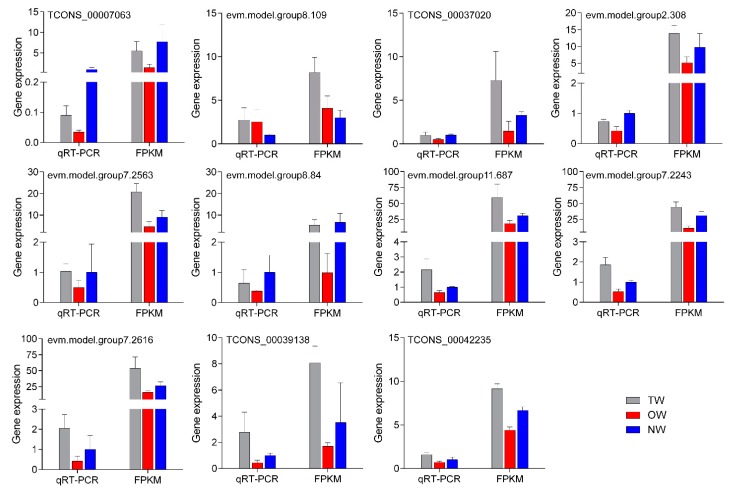
qRT-PCR validation of key genes in different wood types.

To test the reliability of the transcriptome data, eleven mRNAs that were significantly differentially expressed in both comparison groups were selected for qRT-PCR (Figure 10). The results showed that the relative expression trend of the 11 genes in the fluorescence-based qRT-PCR data was consistent with the transcriptome sequencing results. This result indicated that the transcriptome results were highly reliable.

### 2.8. Transcriptional Regulation Network

**Figure 11:**
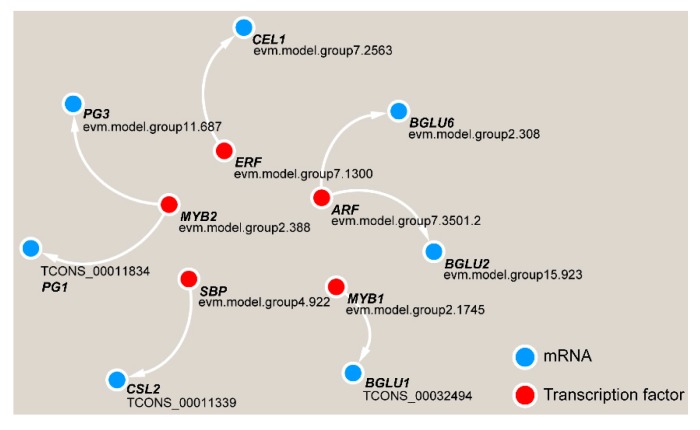
Transcriptional regulatory network in TW formation in *C. bungei.*

Gene function is often inseparable from transcriptional regulation. We analyzed the upstream 2000-bp sequence from the DEG coding sequences and the potential binding motifs of transcription factors, and we found that five transcription factors have paired relationships with key DEGs (Figure 11). Among these transcription factors, the MYB2 transcription factor may regulate *PG*1 and *PG3*, and ARF has a potential targeting relationship with *BGLU2* and *BGLU6*. ERF, SBP and MYB1 may regulate the *CEL1*, *CSL2* and *BGLU1* genes, respectively.

## 3. Discussion

### 3.1. Anatomical Characteristics

A recent report suggested that the G-layer was a part of secondary walls [40]; thus, TW formation could be regarded as secondary wall deposition, which is a unique type of wood formation. Previously, gelatinous fiber was used as a typical feature for the identification of TW, but not all TW maintains a G-layer [9,41]. Fisher and Stevenson [42] and Clair, et al. [8] observed several species of TW under a microscope and found that only 33%~46% of the species of TW had an obvious G-layer. In this study, we also found that the TW of *C. bungei*, which was both naturally and artificially bent, did not have a G-layer. Although some reports have found that the structural characteristics of the secondary wall of TW without a G-layer are similar to those with a G-layer [43], the mechanism and biophysical characteristics of this unique secondary wall deposition method are still poorly understood. We found that TW fibers of *C. bungei* lacked S_3_ layer, it’s consistent with Yoshizawa’s results [9]. He also found anatomical features of *Magnolia* Species TW fiber lacked the S_3_ layer and the S_2_ layer has greater microfibril angles. Maybe this structure is responsible for the high stress of TW.

### 3.2. Cellulose and Hemicellulose Synthesis

The cellulose content in TW is lower than that in OW and NW according to the Raman spectrum (Figure 7), and there are many DEGs involved in cellulose synthesis. Four *CesA* and 2 *CSL* genes are expressed at significantly high levels in TW. High expression of multiple *CesA* genes was found in the TW based on the transcriptomes of poplar, birch and *Eucalyptus* [12,23,34,35], but high *CSL* gene expression was not observed in the corresponding TW. The G-layer is a special cell wall layer that is rich in gelatinous fiber, and high expression of *CesA* may promote cellulose synthesis in the G-layer in trees that have a G layer in TW fiber. In *Arabidopsis thaliana*, *CesA* gene family members have high functional specificity in cell wall synthesis, and only *AtCesA4*, *AtCesA7*, and *AtCesA8* are involved in secondary wall synthesis [44]. In addition, these genes also have strong tissue specificity [45]. In this study, we did not observe the G layer in *C. bungei* TW, and the fiber cell walls were thinner than those of NW. These phenotypes were contradictory to the result that *CbCesAs* genes were highly expressed in TW. It was speculated that these genes were not involved in TW deposition for secondary wall thickening or there were specific protein modifications that make these proteins inactive [39]. Although the CSL and CesA proteins are similar in structure, and increasing number of researchers believe that the *CSL* gene is involved in non-cellulosic polysaccharide synthesis [46,47,48]. This suggests that the significantly high expression of the *CSL* gene in *C. bungei* TW may play a key role in changing the components of non-cellulosic polysaccharides. Moreover, high *XTH* genes and *CSLA9* gene expression was observed in TW of *C. bungei.* It also indicated more non-cellulosic polysaccharides exist in TW. Some studies showed that TW had more xyloglucan which played a key role of adhesion of cell wall layer, generating and transmitting stress [49,50,51]

Furthermore, *CEL* and *BGLU* belong to the cellulose decomposition enzyme system. *CEL* is significantly upregulated in TW, and the product (endoglucanase) potentially has a relationship with cell wall relaxation [52]. It might digest the non-crystalline regions of cellulose microfibrils, resulting in increased wall extensibility and cell growth [10]. *BGLU* was also upregulated in TW, and it encodes a beta-glucosidase protein that hydrolyzes cellobiose. Thus, we hypothesized that the change in the expression of these genes is the main reason for the decrease in secondary wall deposition in TW and prevents G-layer development. At the same time, these enzymes change the cellulose characteristics and may promote the stretching ability of the TW cell wall.

### 3.3. Pectin Synthesis

Pectin is a polysaccharide that is mainly found in the intercellular layer of plants. It induces lignification in plant tissue, enhances plant strength and supports plants [16,53]. The structures of pectins influence their physical-chemical properties [28]. They have been implicated in wall extension and contribute to the mechanical strength, adhesion and stiffness of the cell wall [54]. In our study, one galacturonosyl transferase (*GAUT*) was significantly downregulated, and two *GAUTs* were significantly upregulated in the TW/OW comparison. The synthesis of HG requires the activity of galacturonosyltransferases (GAUTs) [55]. This finding indicates that the pectin type in TW may have changed, as HG pectin has a longer backbone. Gritsch, et al. [6] also found that de-esterified HG was abundant in TW. Moreover, 5 *PG* genes, which encoded polygalacturonase and were associated with the decrease in the pectin content, were highly expressed in TW. Correspondingly, 3 PG proteins were high abundant in TW. However, it also was found that many DEGs were involved pectin degradation in the poplar TW. Some pectinesterases (*PMEs*) were significantly downregulated, while several pectin lyase (*PL*) genes were significantly highly expressed [12], which was different from the results of this study. PL and PG both cleave α-1,4 glucoside linkages, but the former performs trans elimination, while the latter degrades pectin through hydrolysis [56]. However, the expression of pectin lyase was significantly decreased in *Eucalyptus* TW [23,33]. This finding implied that pectin modification played a key role in TW formation, and the specific pectin type determines the specific properties of the TW cell wall [17,18]. We believe that cellulose with high relaxation, the specific pectin type and esterification may affect the TW type and are the basic determinants of the increased tensile stress of the TW of *C. bungei* in response to external stimulation, even though numerous studies have considered the G-layer as the driving force of tensile stress generation in TW [43,57,58].

### 3.4. Transcriptional Regulation in TW Formation

Gene expression is often subject to specific transcriptional regulation, and while there are many modes of regulation at the transcriptional level, the most common mode is regulation via transcription factors. The formation of the cell wall is an extremely complex process, and the transcriptional regulation mechanism of secondary wall formation is relatively clear, exhibiting a multi-level transcriptional regulatory network [1]. In this network, most of the transcription factors belong to the *MYB* and *NAC* gene families, and it has been demonstrated that when overexpressed, these transcription factors are able to trigger the expression of target genes involved in polysaccharide or lignin biosynthesis and promote secondary wall formation [59]. In this paper, we predicted some transcription factors that could regulate the cellulose and pectin degradation genes; these factors not only have a targeted relationship with some key genes but also are co-expressed with target genes. The two MYB transcription factors that were identified in this study may also be part of the secondary wall formation regulatory network. In addition, ERF and ARF are ethylene and auxin-mediated transcription factors, respectively. It is suggested that multiple phytohormones may act as signaling molecules that induce TW formation [60].

## 4. Materials and Methods

### 4.1. Plant Materials

**Figure 12:**
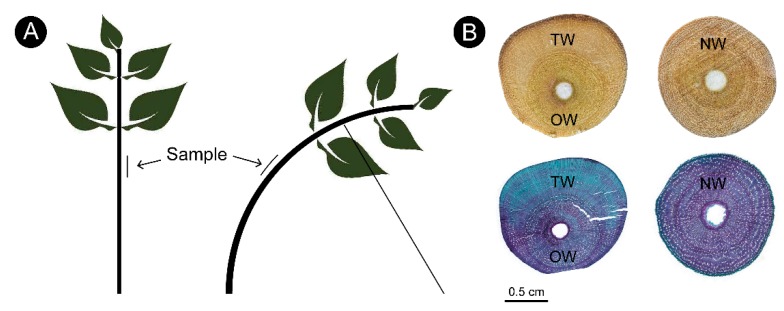
Sample collection for tension wood of *C. bungei.* (**A**) The model of treatment. (**B**) The location of sample collection.

In this study, the clone 8402, which was bred by our research group and has excellent wood physical properties, was selected as the research material. Grafting was carried out at the end of April 2017, and artificial bending was used to induce TW formation in mid-July. The plants were kept in a bent state for 3 months with a consistent angle of 45°. A 5–10-cm sample of the bent stem was taken at a height of 60 cm after removing the bark at this site, and the samples were immediately frozen in liquid nitrogen for preservation. The plants that were not subjected to bending were used as controls, and samples were collected at the same height. Further details of the treatment are shown in Figure 12A.

A section of each wood sample was removed and double-dyed with sarranine and fast green. The tissue dyed blue-green with fast green was TW (Figure 12B). The tissues of each sample were divided according to the staining results of the sections. Five different individual plant samples were mixed into one, and a total of three samples, namely, three biological duplicates, were collected for subsequent experiments.

In March 2019, 9 individual plants that were naturally curved (Figure 1) were selected from the clonal test plantation built by our research group in 2012. The plants were truncated at the bending position, and then, 3–5 samples of 1 cm^3^ xylem were sectioned from the fuzzy area above of the pith and the lower part of the pith.

### 4.2. Anatomy of Xylem

The xylem samples were cut into 1-2 cm segments, and 30 μm slices were prepared by the Leica SM2010 R slicing mechanism. One percent sarranine dye was used for 12 h, and then the samples were successively dehydrated in 30%, 50%, 75%, and 95% ethanol and anhydrous ethanol. Fast green dye (0.1%) was used for 4 min, and the transparency of the slices was achieved with xylene. Finally, slices sealed with Canadian resin were observed and photographed under an optical microscope.

After artificial bending treatment, three individual plants were randomly selected, and 5 slices were selected for each individual plant; five field views of 10- and 100-times magnification were selected for each slice. The length and width of 30 fiber cells were measured in each 100-times magnification field; the number of vessels was counted, and the length and width of the vessels were measured in each 10-times magnification field.

### 4.3. Transmission Electron Microscopy

The xylem samples were cut into small pieces (0.5–1.0 mm^3^) with a sharp blade, placed into 3% glutaraldehyde fixing solution (pH 7.2–7.4), dried under vacuum for 30 min and fixed for 5 h. The samples were washed 8 times with 0.1 mol/L phosphate buffer for 20 min each, fixed at 4 °C for 5 h with 1% osmium acid and washed with double-distilled water 4 times. Then, 30%, 50%, 70%, and 100% ethanol was used for gradient dehydration, and the sample was embedded with Spurr resin at 60 °C. An ultramicrotome with diamond knife was used to slice the sample, which was then stained with lead citrate and uranyl acetate and observed under a transmission electron microscope (HITACHI HT-7700, Tokyo, Japan).

### 4.4. Confocal Raman Spectra and Raman Mapping

The sample measurements were performed with a LabRam Xplora confocal Raman microscope (Horiba Jobin–Yvon, Paris, France) equipped with an Olympus confocal microscope (Olympus, Tokyo, Japan), an oil immersion objective (UPLSAPO 100×oil, Olympus), and a linearly polarized 532 nm laser (Ventus VIS 532, Laser Quantum, Chester, UK), with power set to 8 mW. Spectral data were acquired by the point-by-point scanning microprobe imaging method. The grating was 1200 mm^−1^, the slit width was 100 μm, the numerical aperture was 300 μm, and the scanning step was 0.7 μm. Fifteen spectra were obtained and averaged. The single-point spectral acquisition time was 1 s, and the spectral resolution was 2 cm^−1^. LabSpec6 software was applied to acquire and analyze data. The entire experiment was carried out at a constant temperature (approximately 25 °C)

### 4.5. mRNA Isolation, Library Construction, and Illumina Transcriptome Sequencing

Total RNA of TW, OW and NW was extracted using an RNA reagent kit (DP441; Tiangen Biotech, Beijing, China) according to the manufacturer’s protocol. Three replicates were included for each type of wood. After total RNA was extracted, rRNAs were removed to retain mRNAs. Then, nine transcriptome libraries were constructed for mRNA and small RNA Illumina high-throughput sequencing. The libraries were sequenced using an Illumina HiSeqTM 4000 platform (Illumina, San Diego, CA, USA) by Gene Denovo Biotechnology Co. (Guangzhou, China).

After sequencing, the reads were further filtered to obtain high-quality clean reads according to the following rules: (1) reads containing adapters were removed; (2) reads consisting of all A bases were removed; (3) reads containing more than 10% of unknown nucleotides (N) were removed; and (4) low-quality reads containing more than 50% low-quality (Q-value ≤ 20) bases were removed. rRNA was removed from the reads of each sample, and then the reads were mapped to the reference genome by TopHat2.

### 4.6. Quantification of Transcript Abundance and Differential Expression of mRNAs

Transcript abundances were quantified by RSEM software [61]. The transcript expression level was normalized by using the fragments per kilobase of transcript per million mapped reads (FPKM) method, and the formula is shown as follows, where FPKM (A) is the expression of transcripts A, C is the number of fragments mapped to transcripts A, N is the total number of fragments mapped to reference genes, and L is the number of bases on transcripts A. The FPKM method is able to eliminate the influence of different transcript lengths and amounts of sequencing data on the calculation of transcript expression. Therefore, the calculated transcript expression can be directly used for comparing the differences in transcript expression among samples:

(1) $$\mathrm{FPKM}=\frac{10^{6}C}{NL/10^{3}}$$

The differentially expressed transcripts of coding mRNAs were analyzed. To identify differentially expressed transcripts across samples or groups, the edge R package (http://www.r-project.org/) was used to perform quasi-likelihood F-tests. We identified transcripts with a fold change ≥1.5, and a *p*-value < 0.05, and a FPKM >1 in a comparison as significant differentially expressed genes (DEGs).

### 4.7. Protein Extraction

Total proteins were extracted using the cold acetone method. Samples were ground to powder in liquid nitrogen, dissolved in 2 mL of lysis buffer (8 M urea, 2% SDS, 1×Protease Inhibitor Cocktail (Roche Ltd. Basel, Switzerland)), sonicated on ice for 30 min and centrifuged at 13,000 rpm for 30 min at 4 °C. The supernatant was transferred to a fresh tube. For each sample, proteins were precipitated with ice-cold acetone at −20 °C overnight. The precipitates were washed with acetone three times and redissolved in 8 M urea by sonication on ice. Protein quality was examined by SDS-PAGE (Figure S4).

### 4.8. Protein Digestion and TMT Labeling

Proteins were redissolved in 500 mM TEAB (triethylammonium bicarbonate). Determine the protein concentration of the supernatant using the BCA protein assay, and then transfer 100 μg protein per condition into a new tube and adjust to a final volume of 100 μL with 100 mM TEAB. Add 5 μL of the 200 mM TCEP and incubate sample at 55 °C for 1 h, then add 5 μL of the 375 mM iodoacetamide to the sample and incubate for 30 min protected from light at room temperature. For each sample, proteins were precipitated with ice-cold acetone, and then were re-dissolved in 100 μL TEAB. Then proteins were tryptic digested with sequence-grade modified trypsin (Promega, Madison, WI, USA), and the resultant peptide mixture was labeled with TMT. The labeled samples were combined and dried in vacuum.

### 4.9. High pH Reverse Phase Separation

The peptide mixture was redissovled in the buffer A (buffer A: 20 mM ammonium formate in water, pH 10.0, adjusted with ammonium hydroxide), and then fractionated by high pH separation using Ultimate 3000 system (ThermoFisher Scientific, Waltham, MA, USA) connected to a reverse phase column (XBridge C18 column, 4.6 mm × 250 mm, 5 μm, (Waters Corporation, Milford, MA, USA). High pH separation was performed using a linear gradient. Starting from 5% B to 45% B in 40 min (B: 20 mM ammonium formate in 80% ACN, pH 10.0, adjusted with ammonium hydroxide). The column was re-equilibrated at initial conditions for 15 min. The column flow rate was maintained at 1 mL/min and column temperature was maintained at 30 °C. Twelve fractions were collected; each fraction was dried in a vacuum concentrator for the next step.

### 4.10. Protein Identification and Quantification

Protein identifications were accepted if they could achieve an FDR less than 1.0% by the Scaffold Local FDR algorithm. Proteins that contained similar peptides and could not be differentiated based on MS/MS analysis alone were grouped to satisfy the principles of parsimony. The parameters for the Mascot database search are shown in Table S8. Protein quantification was carried out for the proteins identified in all the samples with unique spectra ≥2. Relative protein quantification was based on the ratios of reporter ions, which reflect the relative abundances of peptides. The Mascot search results were averaged using medians and quantified. Proteins with fold change >1.2 or <0.83 in a comparison and with unadjusted significance level *p* < 0.05 were considered to be differentially expressed.

### 4.11. Functional Enrichment of Differentially Expressed mRNAs and Proteins

Differentially expressed coding RNAs were then subjected to Gene Ontology (GO) function and Kyoto Encyclopedia of Genes and Genomes (KEGG) pathway enrichment analyses. All DEGs were mapped to GO terms in the GO database (http://www.geneontology.org/), and gene numbers were calculated for every term. The GO terms significantly enriched in DEGs compared to the genome background were defined by a hypergeometric test. The formula for calculating the *p*-value is as follows:

(2) $$P=1-\overset{m-1}{\underset{i=0}{\sum }}\frac{\left(\begin{array}{c} M\\ i \end{array}\right)\left(\begin{array}{c} N-M\\ n-i \end{array}\right)}{\left(\begin{array}{c} N\\ n \end{array}\right)}$$

Here, *N* is the total number genes with GO annotation; *n* is the number of DEGs in *N*; *M* is the total number of genes annotated to specific GO terms; *m* is the number of DEGs in *M*. The calculated *p*-values were subjected to FDR correction, taking FDR ≤ 0.05 as a threshold. GO terms meeting this condition were defined as significantly enriched GO terms in DEGs. This analysis was able to identify the main biological functions of the DEGs.

KEGG is the primary public pathway-related database [62]. Pathway enrichment analysis identifies metabolic pathways or signal transduction pathways significantly enriched in DEGs compared with the whole genome background. The formula used to calculate the *p*-value was the same as that used in the GO analysis.

Proteins were also annotated against the GO and KEGG databases for functional identification. Significant GO functions and pathways were examined for the differentially expressed proteins (DEPs) with *p* values ≤ 0.05.

### 4.12. Quantitative Real-Time (qRT)-PCR

The 7500 Real-Time PCR System (Applied Biosystems, Foster city, CA, USA) and TB Green Premix Ex Taq™ (TaKaRa, Dalian, China) were used to perform qRT-PCR. The 2^−ΔΔCt^ method was used to calculate relative expression levels. Cbu-actin was amplified as the endogenous control [63]. The detailed primer information is shown in Supplementary Table S9.

### 4.13. Data Deposition

The transcriptome sequence data have been deposited to the NCBI via Sequence Read Archive (SRA) with the accession numbers: PRJNA559964.

## 5. Conclusions

Previous studies initially revealed changes in gene expression during TW formation through transcriptomics or proteomics. Our research, based on these studies, combined transcriptional and proteomic information to further accurately uncover the key functional genes/proteins and regulators involved in TW formation. We found that there was no obvious G-layer in the TW fiber of *C. bungei*. These results will also provide insight into TW formation in other tree species. The cellulose and pectin polysaccharide metabolic pathways were examined, and the transcription and protein expression levels between TW contain a G-layer and TW lacking a G-layer were studied; we found some differences in TW formation in *C. bungei* in the cellulose and pectin metabolic pathways. Meanwhile the potential transcription factors involved in regulating polysaccharide metabolism were also predicted. This research will serve as a roadmap for studying the mechanisms underlying xylogenesis and TW formation and will aid the genetic improvement of wood properties.

## Supplementary Materials

Supplementary materials can be found at https://www.mdpi.com/1422-0067/21/5/1686/s1.
