# Supplementary material for: Transcriptomics and Proteomics Reveal the Cellulose and Pectin Metabolic Processes in the Tension Wood (Non-G-Layer) of Catalpa bungei
Source: Int J Mol Sci. 2020 Mar 1;21(5):1686. doi: 10.3390/ijms21051686 (PMC7084593; doi:10.3390/ijms21051686)
Supplement: Supplementary file 1 [file ijms-21-01686-s001.zip › Supplementary_Figure.pdf]

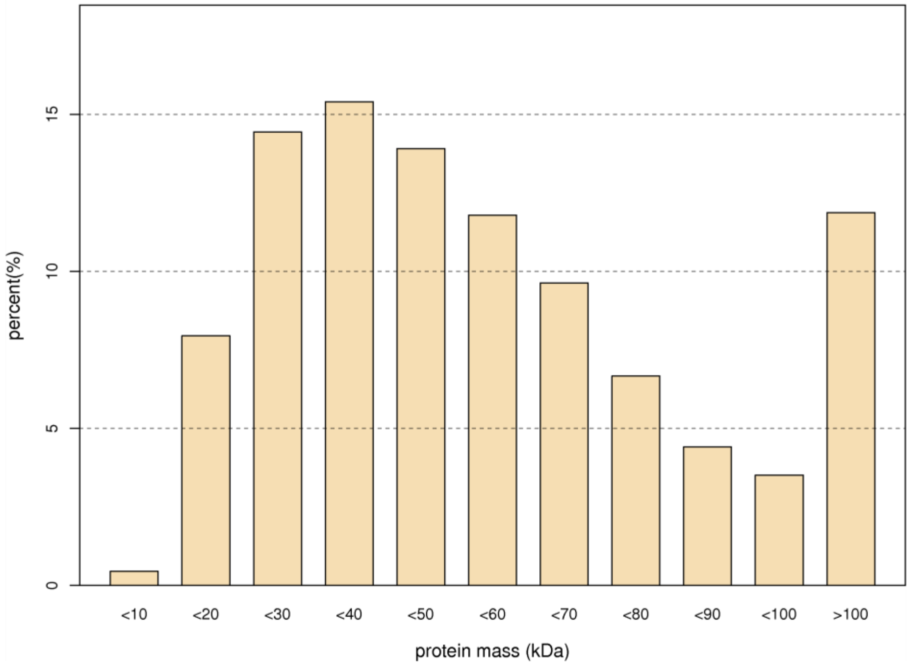

Supplementary Figure 1. The mass distribution of protein

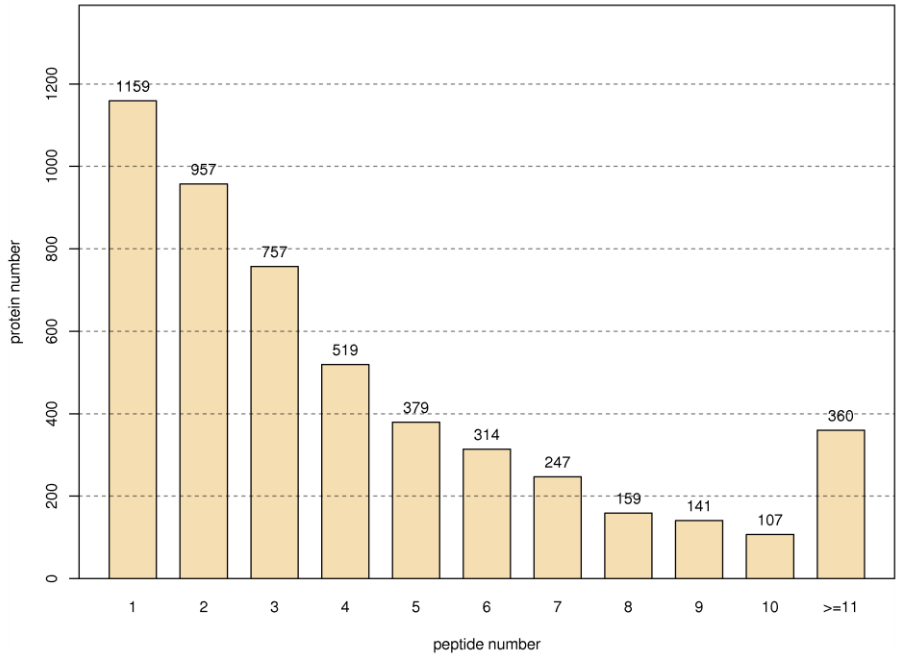

Supplementary Figure S2. The Peptide length profile.

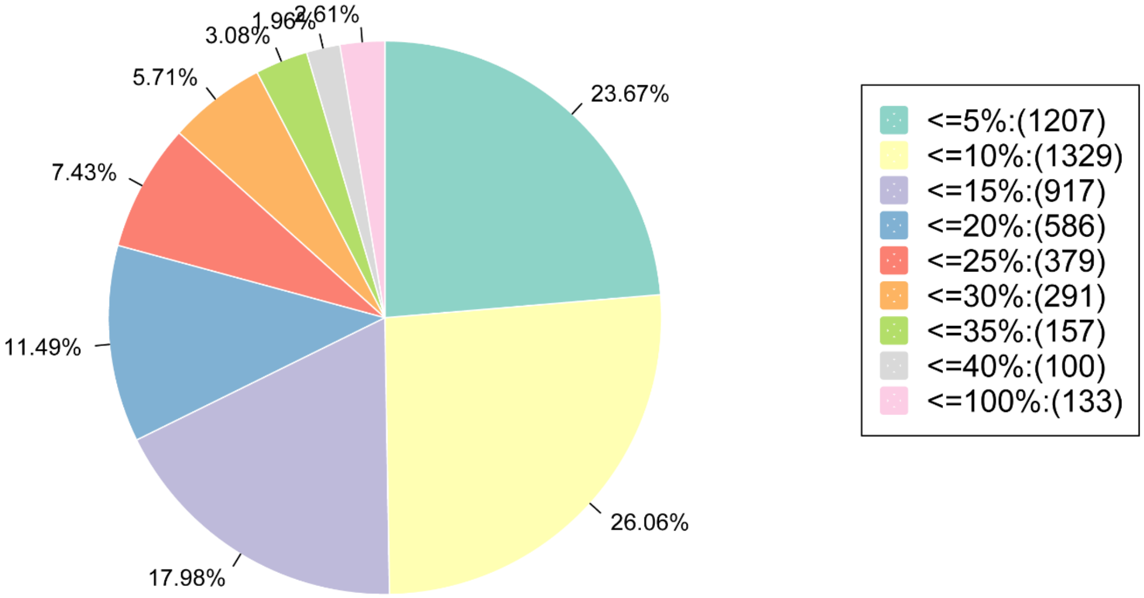

Supplementary Figure S3. Distribution of protein's sequence coverage.

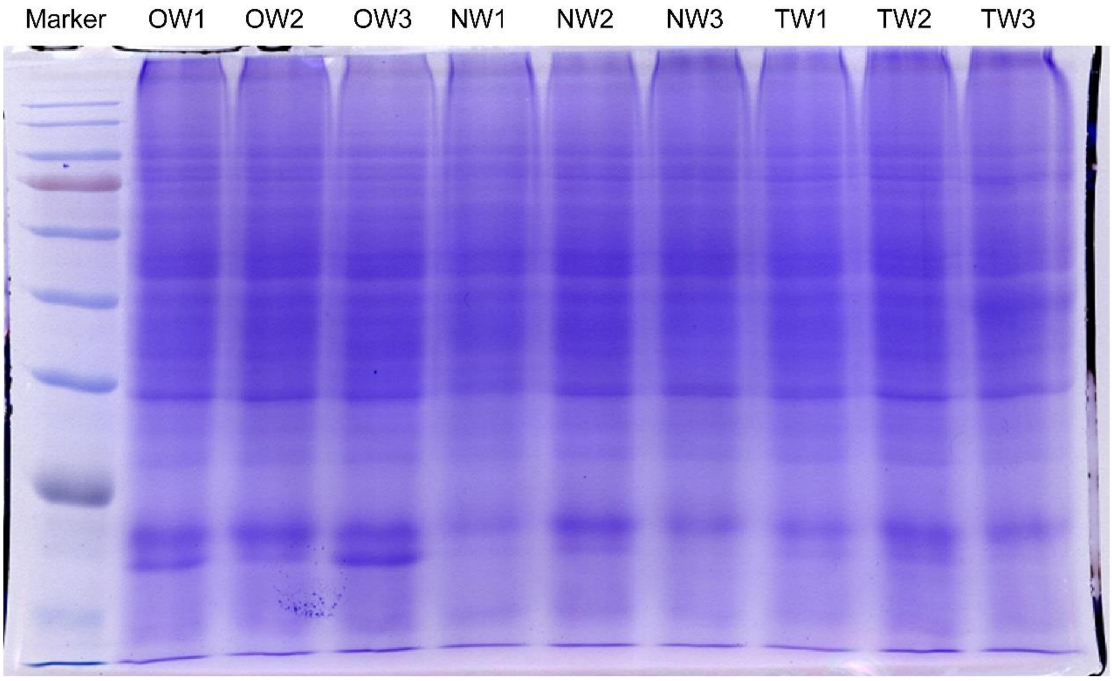

Supplementary Figure S4. The SDS-PAGE map of TW，OW and NW of *C. bungei*.
